# Severe psychological impact and impaired quality of life after a spontaneous haemoperitoneum in pregnancy in women with endometriosis and their partners

**DOI:** 10.52054/FVVO.13.2.021

**Published:** 2021-06-28

**Authors:** A.M.F. Schreurs, M.C.I. Lier, D.B.M. Koning, C.W.A. Brals, M.A. De Boer, C.B. Lambalk, M De Wit, V Mijatovic

**Affiliations:** Amsterdam UMC, Vrije Universiteit Amsterdam, Endometriosis Center, Department of Reproductive Medicine, Amsterdam Reproduction and Development research institute, de Boelelaan 1117, Amsterdam, the Netherlands;; Amsterdam UMC, Vrije Universiteit Amsterdam, Department of Medical Psychology, de Boelelaan 1117, Amsterdam, the Netherlands; Amsterdam UMC, Vrije Universiteit Amsterdam, Department of Obstetrics and Gynaecology, de Boelelaan 1117, Amsterdam, the Netherlands

**Keywords:** Quality of Life, Quality of Care, haemoperitoneum, obstetrics, endometriosis

## Abstract

**Background:**

Spontaneous Haemoperitoneum in Pregnancy (SHiP) is a rare, but life-threatening complication of pregnancy that occurs predominantly in the third trimester of pregnancy and is associated with adverse pregnancy outcomes. Recently the largest case series in literature was published describing 11 Dutch cases of SHiP in women with endometriosis.

**Purpose:**

To investigate experiences, psychological impact, and quality of life after SHiP.

**Methods:**

A mixed-methods study was performed in women with a history of SHiP and their partners, including all known cases in the Netherlands between 2007 to 2015. Semi-structured in-depth interviews were organized between 2016 and 2017 and analysed thematically with a framework approach. Participants were asked to complete questionnaires investigating the impact of the event (Impact of Event Scale) and Quality of Life (RAND-36).

**Results:**

Out of a total of 11 known cases, 7 women agreed for be individually interviewed. From these, all women described a freeze response at the moment of SHiP, combined with either an anxious reaction or a survival mode mind-set. All women received psychological help after SHiP. Still, the feeling of not being heard by the medical staff was present in all women. Other themes such as postpartum period, bonding with their child, effect on daily life, reviving the event, and future pregnancies were also identified in the interviews. In regard to their partners, 3 were interviewed, hence no saturation was achieved. Finally, the questionnaires showed lower Quality of Life and an impact score of ≥ 8/10.

**Conclusion:**

SHiP had a profound impact on women and their partners. Dedicated psychological help should be offered to all women after experiencing SHiP.

## Introduction

Spontaneous Haemoperitoneum in pregnancy (SHiP) is a rare, non-traumatic intraperitoneal bleeding that occurs in pregnancy and up to 42 days postpartum, requiring surgical intervention or embolization (Schaap et al., 2019). This definition excludes ectopic pregnancy, uterine rupture and caesarean section associated bleeding as causes of SHiP. The aetiology of SHiP is still unknown, but two risk factors are currently identified: endometriosis and conceiving after artificial reproductive techniques such as IVF (Brosens et al., 2016; Lier et al., 2017). A previously published systematic review showed that all known biopsies of the bleeding site found decidualisation, even if there were no macroscopic signs of endometriosis (Brosens et al., 2016).

First clinical signs of SHiP are sudden onset of abdominal discomfort and pain, followed by decreased haemoglobin levels and signs of hypovolemic shock when haemorrhage is severe or diagnosis delayed. This can result in severe complications for both mother and child. From literature maternal shock, emergency surgery, caesarean section and hysterectomy have been reported (Lier et al., 2017; Brosens et al., 2009; Ginsburg et al., 1987). For the foetus, SHiP can result in foetal distress, foetal demise, iatrogenic preterm birth, and admittance to the Neonatal Intensive Care Unit (Lier et al., 2017). In last decades SHiP associated maternal mortality has nearly disappeared due to improved recognition and management of SHiP. However the perinatal mortality rate is still high reaching up to 27% of reported cases (Lier et al., 2017).

Severe pregnancy complications could have a significant psychological impact, although findings are mixed and require further research. One study found that up to six years postpartum, 28% of women with a major obstetric haemorrhage still reported a negative effect of the haemorrhage on their lives with 23% of the cases abandoning their desire for a successive pregnancy (de Visser et al., 2018). In contrast, van Stralen et al. (2018) found no difference in quality of life scores in women treated for major obstetric haemorrhage in comparison to reference groups.

In 2017, Lier et al. published the largest case series of SHiP known in literature including eleven women all having a history of endometriosis (Lier et al., 2017). The researchers noticed the psychological impact that SHiP events had on women and their families. Therefore, it was hypothesized that severe pregnancy complications and emergency surgeries following SHiP may result in psychological complaints and a decreased quality of life in women experiencing SHiP as well as in their partners. Consequently, the aim of this study was to investigate the experiences of women and their partners with a SHiP event and to determine the psychological impact of a SHiP event.

## Materials and methods

### 

An observational study combining qualitative and quantitative research methods to investigate experiences, identify the psychological impact and determine the quality of life after a SHiP event was performed in 2016 and 2017. Qualitative methods were used primarily to investigate experiences and psychological impact. Due to the exploratory aim of the study, a phenomenological approach was used with a constructivist paradigm. These qualitative methods were subsequently combined with quantitative questionnaires to investigate quality of life. All women and their partners from a previous case series of SHiP events were invited to participate in this study (Lier et al., 2017). Ethical approval was granted by the Institutional Review Board of Amsterdam UMC, Vrije Universiteit Amsterdam (METc VUmc 2016.236). As the study aimed to gain insight in women’s personal experiences, women and their partners were invited for a semi- structured face-to-face in-depth interview after signed informed consent and asked to complete two questionnaires. By choosing one-on-one interviews as a research method, participants were stimulated to focus on their own experiences without external influences.

### Interviews

The interviews were conducted by an independent medical psychologist, trained and experienced in conducting semi-structured interviews. The medical psychologist was independent and had no previous contact with participants. As a guide for the semi- structured interviews, five questions related to the SHiP event were used (Appendix A and B). The questions were designed to aid the participant to discuss personal experiences with the SHiP event. First, participants were stimulated to recall the event. Then, the period directly after the SHiP event and the recovery period were discussed. Finally, psychological impact and need for help were discusses.

All interviews were audiotape recorded and transcribed verbatim. Data saturation of information was pursued, signifying that no new information was heard during the later interviews and that further data collection is not necessary. Except for one interview that was conducted using video call due to emigration abroad, all interviews took place in one tertiary hospital. This hospital was the treating centre during the SHiP event for 4 women.

Interview analysis was conducted using thematic analysis, allowing the researcher to start analysis with concept themes (build on the sub-questions) and build new themes after they emerge in the codes (Braun and Clarke, 2006). Interview analysis was performed by independent researchers with no previous contact with the participants and no involvement in the cases in the past.

### Questionnaires

The validated Dutch version of the Impact of Event Scale (IES) is a self-reported questionnaire assessing subjective distress after a serious life event (Horowitz, 1979; Brom and Kleber, 1985). The IES contains 15 items divided into two dimensions of psychological adaptation: ‘avoidance’ and ‘intrusion’, with higher scores indicating more distress. Scores were compared to reference groups that included women from a stress clinic awaiting psychotherapy (van der Ploeg et al., 2004) and Dutch individuals exposed to work- or war related traumas or disasters (Horowitz et al.,1979).

The Dutch RAND-36 is a widely used, highly validated tool that measures experienced health and health-related quality of life (Ware and Sherbourne, 1992; VanderZee et al., 1996). Outcomes can be scored on a scale of 0 – 100, with a higher score correlating with a better quality of life. Scores were compared to gender and age reference groups (Van der Zee and Sanderman, 2012).

### Statistical analysis

SPSS version 22 (IBM) was used for analysing descriptive data. Continuous data were presented as mean and standard deviation when data was normally distributed, and as medians and ranges by non-parametrical data. All questionnaires were analysed through descriptive analysis due to the small sample size.

## Results

### 

**Figure 1:**
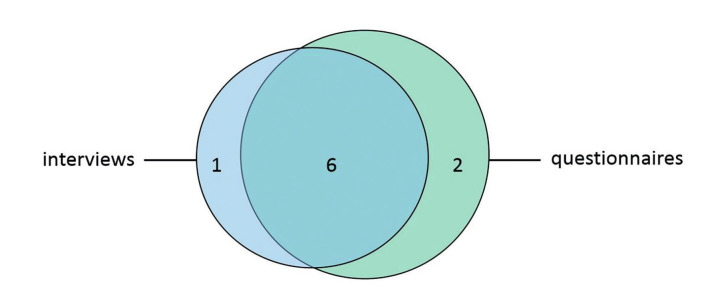
— Overview of included women 7 women agreed to the interviews and 8 women completed the questionnaire. Of the included women, 6 agreed to both the interviews and the questionnaires.

All 11 women with a history of SHiP and their partners that were included in the study by Lier et al. were asked to participate (Lier et al., 2017). In total 7 women agreed to the interview and 8 completed the questionnaires. Of these participants, six women completed both the interviews and the questionnaires. One woman only participated in the interview and two women solely completed the questionnaire (figure 1). Six of the partners completed the questionnaire, of which 3 also agreed to participate in the interviews. Reason for refusal to participate in either were being too busy and finding it emotionally too stressful to discuss the SHiP event again. Three out of 11 partners agreed to participate in the interviews and in total six partners completed the questionnaires. Participants’ characteristics can be found in Table I. All women experienced the SHiP event in the Netherlands between 2007 and 2015. No maternal or perinatal mortality occurred in this case-series.

**Table I:** Baseline characteristics of included women.

Case	Recurrent SHiP	Age at SHiP (y) Parity (G, P)	Endometriosis (rASRM stage)	GA at moment of SHiP (wk+d)	Mode of conception	Estimated blood loss (ml)	GA delivery (wk+d)	Time since SHiP at moment of interviewing (y)	Interviews and/or questionnaire
A		38 G3P0	Yes (IV)	19+3	IVF	3000	39+0	2	I
B		35 G1P0	Yes (IV)	28	IVF	1100	28+5	2	Q†
C		34 G3P2	Yes (IV)	23+2	Natural conception	1000	-	5	I†+Q†
	Recurrent	“	“	24+3		Unknown	35+5		
D		33 G1P0		34+2	Natural conception	600	34+2	2	Q
	Recurrent	33 G1P1	Yes (IV)	pp+12		2000	-		
E		37 G4P1	Yes (IV)	40+5 (labour)	Natural conception	Unknown	40+5	6	I+Q†
	Recurrent	37 G4P2	“	pp+30		3000	-		
F		28 G1P0	Yes (IV)	37+6 (labour)	IVF	100	37+6	5	I+Q
G		37 G5P2	Yes (unknown)	21	Natural conception	2000	37	3	I+Q†
H		31 G2P0	Yes (II)	33+5	Natural conception	3000	33+5	9	I†+Q†
I		37 G2P0	Yes (IV)	30+1	IVF	1750	30+1	1	I†+Q†

ART, artificial reproductive technology; d, day; G, gravida; GA, gestational age; IVF, In Vitro Fertilization; ml, milliliters; no, number; P, para; pp, postpartum; rASRM, revised American Society for Reproductive Medicine;wk, week; y, years;

† Partner also participated

### Interviews – Women

#### 

Saturation was achieved in the semi-structured interviews. From the interviews seven themes were identified: 1) initial response, 2) postpartum period, 3) support, 4) bonding with their child, 5) daily life, 6) reviving the event, and 7) future pregnancy.

#### Initial response

The SHiP event was described as a painful event with a sudden onset by all women. Four women immediately thought something was wrong.

“This is not right, something must be really wrong”

Three women predominantly wanted to get rid of the pain.

“Please remove this pain, whatever the end is”

All women described a freeze-like response (Bracha, 2004, Schmidt et al., 2008), but they experienced this in two different ways: anxious or without fear. The women that described being anxious were afraid that they would die.

“Should I write a farewell letter?”

“I really had a mortal fear. I really thought I would never get out of it. Yes, it was really heavy”.

The women that did not mention fear, seemed not to be aware of the possible severe consequences of the situation they were encountering and consequently they may have suppressed their emotions.

“Don’t think about emotions, it [SHiP event] is over”

“I… had the idea that I was in a survival mode”

A minority of women described fear of losing their child.

“What I remember most is… pure fear, the fear for my babies”

#### Postpartum period

SHiP did negatively influence the postpartum period for three women. They experienced it as a tough and negative period in which they could not fully enjoy their new-born child since they were still busy recovering.

“I had the feeling that I always was one step behind”

and

“I was actually too busy with myself to have room for my child”

In one of these women the SHiP event occurred during the second stage of labour, the other two women had a caesarean section under full anaesthesia. Four women experienced their post- partum period, despite the SHiP event, as a positive period.

“It all worked out well. We are both still alive. I was very pleased”

#### Support

All women described that they did not feel heard by the medical staff during their SHiP event.

“I was dying, but she [nurse] did not know that at the time. It is totally underestimated, not acknowledged, not taken seriously”.

Women described that they missed a leading health care professional and its reassurance during their admittance.

“What I found the worst, was that no one was my trustee / person of trust”

and

“No one, no one that took control. I had no idea who my doctor was”

When looking at the support that women received from the medical staff we found that all women eventually received psychological help after the SHiP event. This was either given by a social worker or a psychologist. None of the women reported current psychological problems related to their SHiP event and none received psychological help at the moment of interview.

For one woman the consequence of the experiences with SHiP was that she lost her trust in doctors:

“I go to the GP more often now and I have less faith in doctors”

She also mentioned she listens more to her intuition now than before the SHiP event. For another woman the SHiP event had the consequence that she wants to keep control over things in life, after experiencing having no control during the SHiP event. She sought psychological help to cope with this. A third woman revealed she lost a bit of joy in life after the event.

All women mentioned they had a supportive partner during and after the SHiP event. However, one woman mentioned she was not in tune with her partner as she was still recovering while he was focusing on the fact that the danger as caused by the SHiP event was evaded.

“My husband always said when someone was calling: Oh, she is doing O.K.. And I was like: F***, I am not O.K., stop saying I am O.K.“

#### Bonding with child

Six women reported they immediately felt attached to their child.

“When they put him on my skin, he was my baby forever”

One woman felt no attachment to her child as she was still busy recovering from the SHiP event herself.

“I didn’t even look at my child… I was not attached to him”.

After a few months, she started to create a bond with her child, but she explained she had to work hard to achieve that. Another woman explained that it took time to get to know each other. For this woman the SHiP event had led to an emergency caesarean section under full anaesthesia. Her child was born premature and was immediately admitted to the neonatology ward.

“I experienced it as unreal, you do not have a normal delivery, you suddenly have a child”.

At moment of interviewing, all women seem to have bonded adequately with their child and they all expressed their love for them.

#### Daily life

SHiP had a major impact on daily life after the event; two women quitted their jobs and two other women did not work for almost one year.

“I decided to quit my job, I just could not do it anymore”

One woman works less now than before the event;

“I want to spent as much time as possible with my child”

Another woman started to work after the event, but physically collapsed after a while and had to renounce from work for another month.

Amongst all women undergoing SHiP, three women were temporarily limited in daily activities after the event.

“I could not do anything, I could not even go for a walk. I could not ride a bike”

Two women are still having physical limitations because of the SHiP event at moment of interviewing. They are both tired quickly.

“…I need to lie down on the sofa, not because I want to, but I have to, to get through the day”.

Both women are unsure if this is a consequence of the SHiP event or because of the endometriosis. In addition to the negative impact of SHiP on daily life, the event developed a new perspective on the important things in life (benefit-finding) for two women.

“I am extremely positive now. I am not going to miss anything. I want some quality time with the people I like, I love.”

Both women appreciate small things in life more than they did before the SHiP event.

#### Reviving the event

None of the women reported nightmares concerning the SHiP event. One woman revives the event annually on her child’s birthday, although this becomes less intense every year. Abdominal pain reminds one woman of the SHiP event and the hospital brings up memories for another woman. A third woman described a temporary resistance towards hospitals.

“Every time I passed the [HOSPITAL] I got a stomach ache, I had a knot in my stomach.”

Two women described they cannot watch movies with a specific topic (morphine administration or a documentary about the neonatology ward) due to the memories that arise when watching these.

“It brings me back into the position where I was so weak… I start feeling sick”

#### Future pregnancy

In one case the SHiP event had affected plans with regard to having more children.

“If it [SHiP] happens again, I die… I don’t have the energy to fight again”.

For two women their wish for a next pregnancy would have changed if SHiP had occurred in their first pregnancy. SHiP did not affect plans with regard to having children for three women, two of them were undergoing fertility treatment at moment of interviewing. One woman conceived again after the SHiP event. She had an uneventful successive pregnancy.

### Interviews – Partners

No saturation was achieved in the interviews with the partners. All three participating partners were male. One partner described a freeze response, similar to the women:

“You are looking from the side-line” and “I just had to follow … you let everything happen”

This partner did not have any troubles recovering from the SHiP and did not express a psychological impact. The second partner described the SHiP event as the most intense hours in his life, he experienced it as a daze where minutes felt like hours. The third partner described that he physically collapsed after the SHiP event was averted. Overall no recurrent themes were identified as the three partners described three different experiences during the SHiP event.

### Questionnaires

**Figure 2:**
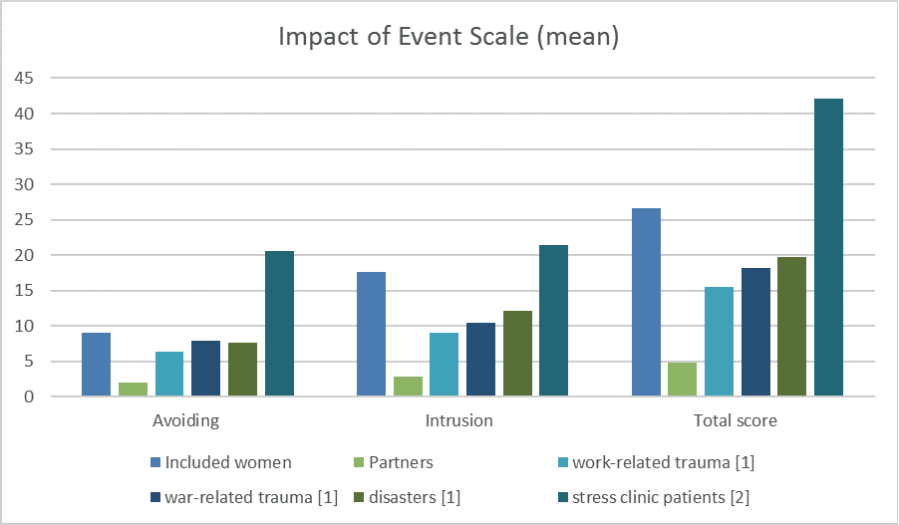
— Impact of Event Scale [1] Horowitz et al. 1979 [2] van der Ploeg et al. 2004

When asked to score the impact of the event on a scale from 0 to 10, with ten representing the highest impact, all women scored the impact of the SHiP event on their lives with a score of eight or higher. Four women ranked the SHiP event at ten. In figure 2 the results from the Impact of Event Scale in our women and their partners are compared to those of Dutch reference groups. No comparative statistics could be performed due to the small sample, but it seems that our participants scored higher on ‘avoiding’ compared to Dutch people experiencing work-, war related trauma’s and disasters, but not as high as women awaiting psychotherapy after a serious life event. This was also the case for ‘intrusion’. Partners, on the other hand, scored low on the IES and showed little signs of avoidance or intrusion.

**Figure 3:**
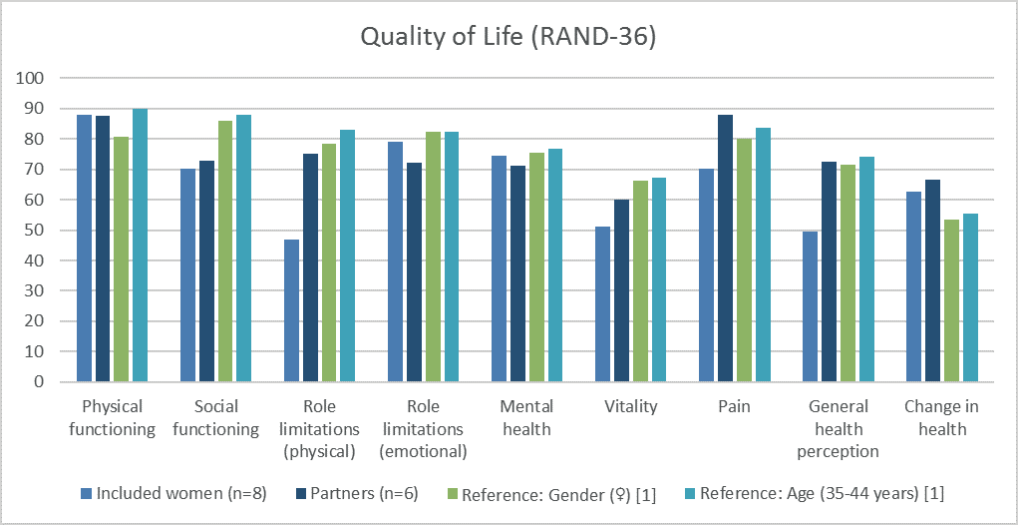
— Quality of Life as measured with the RAND-36 [1] van der Zee et al., 2012

Figure 3 shows the quality of life scores as measured with the RAND-36 for the women and their partners compared to women from the general populations and compared to age controls (VanderZee and Sanderman, 2012). Participants seem to score worse on the domains social functioning, physical role limitations, vitality and general health perception. Partners likewise scored worse on social functioning and vitality.

## Discussion

### 

This study is an additional investigation of the largest known case-series on women experiencing SHiP (Lier et al., 2017). It is, to our knowledge, the first study on the psychological impact and quality of life after a SHiP event. By using both, questionnaires and interviews, the impact of a SHiP event could be analysed from different angles. This study reports that SHiP had a profound impact on the daily life of these women. All women received psychological help and lower quality of life scores were reported post SHiP.

### Interviews

Based on the semi-structured interviews, better support during the event should be given by healthcare providers for all women and especially those who express anxiety and fear of death. This is in line with previous research indicating a lack of information, acknowledgement and reassurance in women with major obstetric haemorrhage (Thompson et al., 2011, de Visser et al., 2018, Sentilhes et al., 2011). Another population of women with major obstetric haemorrhage also reported long term negative effects of the event and refraining from a future pregnancy due to fear of recurrence (Sentilhes et al., 2011). Gottvall et al. previously described the associated between a negative birth experience and fewer subsequent children and a longer interval between children conception (2002). This is similar to views on future pregnancies as found in the current study.

Considering bonding, all women reported secure attachment to their child on the long term. However, immediately after the SHiP event, some women reported difficulties in bonding with their child. This could be due to maternal stress and maternal pain, as these aspects were identified to negatively influence mother-child bonding in previous research (Kinsey et al. 2014). Another explanation might be that some of the children were admitted to the neonatal ward, as separation from your child after birth can cause more concern and less feelings of attachment (Feldman et al., 1999).

The physical fatigue described by participants as a long-term consequence could possibly be explained by the presence of endometriosis as fatigue is associated with endometriosis (Dunselman et al., 2014). On the other hand, the fatigue could be a long-term complication of SHiP too as fatigue was also described as a long term side effect of major obstetric haemorrhage (de Visser et al. 2018).

### Quality of Life

Previous research among women with major obstetric haemorrhage showed no signs of diminished quality of life (van Stralen et al., 2018, van Stralen et al. 2010). However, all participating women in our study scored worse on quality of life after a SHiP event compared to age- and gender reference groups. Previous studies have shown that endometriosis negatively influences quality of life (Culley et al., 2013, Gao et al., 2006; Young et al., 2015). Endometriosis may therefore act as a confounder, as all participants are diagnosed with endometriosis.

### Impact of the event

Women scored relatively high on the Impact of Event Scale compared to a previous study conducted in the Netherlands (van der Ploeg et al., 2004). This emphasizes that experiencing a SHiP event is a serious event with possible severe impact on the women undergoing SHiP. Caution has to be taken as we compare our participants to reference groups from both genders and different age groups. Furthermore, it seems that in general, women score higher on the IES compared to men (Horowitz et al., 1979). Due to the limited number of participants we were not able to test for validity and reliability. Nevertheless, these results show that a SHiP event is a serious event that requires psychological support.

### Partner

Unfortunately, not all partners participated in the interviews and we were not able to achieve saturation in the in-depth interviews. As we do not know the reasons for refraining from participation in the partners, we were not able to investigate participation bias. Although we did not reach saturation, we did find that a SHiP event had a severe impact on the male partner as well. As a bystander it can be emotionally difficult to see your partner suffering, as previously described in partners of women with a post-partum haemorrhage (Dunning et al., 2016). In such partners, higher scores on post-traumatic stress were described compared to controls, even though none of the partners were eventually diagnosed with PTSD (van Steijn et al., 2020). Furthermore, van Stralen et al. found no diminished quality of life in male partners after haemorrhage post-partum (2018). The diminished quality of life findings in male partners of women with a SHiP event, as reported in this current study, could also be due to the fact that all women were are also diagnosed with endometriosis. Culley et al. previously reported that male partners of women with endometriosis report a negative impact on emotional well-being (2017). Given the range of experiences of the men interviewed in our study, the results call for further research in partners.

### Strengths and limitations

Despite the small sample size, saturation was achieved in the in-depth interviews in the women with SHiP. However, due to this small sample size, no comparative statistical analysis could be performed for the questionnaires. Furthermore, even though the interviewer summarized and asked for more clarity when needed, member checking would have increased credibility and validity. Member checking, or respondent validation, is a tool to evaluate the collected information of a qualitative study by allowing participants to provide feedback on the findings. Although current guidelines advise to use member checking, the true added value is debated (Birt et al., 2016; O’Brien et al., 2014; Tong et al., 2007).

Triangulation of data was sought after by using both qualitative and quantitative research methods; women where not only qualitatively interviewed on their traumatic events, this was also quantified by using questionnaires. A helpful addition and new point of view might have arisen from interviewing health care providers on their experiences of the SHiP events. Currently, the Netherlands Obstetric Surveillance System (NethOSS) is auditing all registered SHiP cases in the Netherlands between April 2016 and April 2018. A team of experienced health care providers will audit these cases and describe health care providers’ point of view on the cases.

### Clinical implications

In previous research, risk factors for developing post-traumatic stress disorder included emergency caesarean sections, severe labour pain and sudden complications in pregnancy, such as preeclampsia and preterm birth (Stramrood et al., 2011; Stramrood et al., 2011). Most of these risk factors are present in women with SHiP. We therefore advise physicians that treat women udergoing a SHiP event, to pay considerable attention to the psychological impact of the event and to offer post-event psychological help to all treated women. All participating women described not feeling heard at time of SHiP event and/or during the subsequent period. This might be addressed by listening carefully to the women and appointing her to a single leading healthcare professional. Patient-reported outcome measures (PROMS), such as a health-related quality of life questionnaire, could be helpful in assessing the patient perspective in a systematic way. These measures could further play an important role in identifying patients’ needs in clinical encounters, improving communication and decision-making with health care professionals, and making care more patient-centred (Ishaque et al., 2019).

Even though we were unable to fully analyse the impact on the partners, our results suggest an important impact of a SHiP event on them. We therefore advice to pay attention to the partners as well. Involving them during the event (with information) and after the event (psychological help) might contribute to their overall wellbeing and aid capacity towards their partner.

## Conclusions

SHiP has a profound impact on live after SHiP, although women respond to the SHiP with different reactions. Caregivers should tailor their care by using the experiences as described in the current study. Tailored care may contribute to appropriate counselling concerning the psychological impact of SHiP and could lead to a more extensive follow up to recognize the need for psychological help, although the researchers believe that psychological help should be offered to all women directly after SHiP already.

## Appendices

### Appendix A

Questions used for semi-structured interviews

1. You experienced a major bleeding during or right after your pregnancy, what do you remember?
2. How much impact did the event have on you? Score it on a scale from 0 – 10.
3. How did you experience the period right after the bleeding?
4. How was your recovery after the bleeding? Were there any psychological problems?
5. - Did you have the need for psychological help?
  - If yes, did you seek for psychological help yourself or was it offered to you and which type of help did you receive?

### Appendix B

Questions used for semi-structured interviews with partners

1. What do you remember of the period you partner had a major bleeding during or right after her pregnancy?
2. How much impact did the event have on you? Score it on a scale from 0 – 10.
3. How did you experience the period right after the bleeding?
4. How have you been after the bleeding? Were there any psychological problems?
5. - Did you have the need for psychological help?
  - If yes, did you seek for psychological help yourself or was it offered to you and which type of help did you receive?
